# Trional in Delirium Tremens

**Published:** 1894-08

**Authors:** 


TRIONAL IN DELIRIUM TREMENS.—Dr. Russell Bellamy, in treating ten cases of delirium tremens with trional, a new hypnotic, records the results of his work in the *New York Medical Journal*, as follows:

1. Delirium was controlled with greater rapidity and safety by trional than by other hypnotics.

2. In the majority of cases a marked stimulant effect was observed, possibly on account of the methylic and ethylic elements which enter into the composition of the drug.

3. On account of the low temperature noted in all cases, trional must possess antipyretic properties, thereby simulating its allies of the phenol group.

4. It was always well borne by the stomach, and in one case was rapidly absorbed when administered per rectum.

5. No unpleasant after-effects were observed, and in all cases, except one, and a tuberculosis complication, recovery was speedy.—*Exchange.*

